# Is adding maternal vaccination to prevent whooping cough cost-effective in Australia?

**DOI:** 10.1080/21645515.2018.1474315

**Published:** 2018-06-22

**Authors:** Laure-Anne Van Bellinghen, Alex Dimitroff, Michael Haberl, Xiao Li, Andrew Manton, Karen Moeremans, Nadia Demarteau

**Affiliations:** a CHESS in Health, Bonheiden, Belgium; b GSK, Melbourne, Australia; c GSK, Wavre, Belgium; d HEARS Pty Ltd, Melbourne, Australia

**Keywords:** Australia, cost-effectiveness, maternal vaccination, pertussis, whooping cough

## Abstract

Pertussis or whooping cough, a highly infectious respiratory infection, causes significant morbidity and mortality in infants. In adolescents and adults, pertussis presents with atypical symptoms often resulting in under-diagnosis and under-reporting, increasing the risk of transmission to more vulnerable groups. Maternal vaccination against pertussis protects mothers and newborns. This evaluation assessed the cost-effectiveness of adding maternal dTpa (reduced antigen diphtheria, Tetanus, acellular pertussis) vaccination to the 2016 nationally-funded pertussis program (DTPa [Diphtheria, Tetanus, acellular Pertussis] at 2, 4, 6, 18 months, 4 years and dTpa at 12–13 years) in Australia.

A static cross-sectional population model was developed using a one-year period at steady-state. The model considered the total Australian population, stratified by age. Vaccine effectiveness against pertussis infection was assumed to be 92% in mothers and 91% in newborns, based on observational and case-control studies. The model included conservative assumptions around unreported cases.

With 70% coverage, adding maternal vaccination to the existing pertussis program would prevent 8,847 pertussis cases, 422 outpatient cases, 146 hospitalizations and 0.54 deaths per year at the population level. With a 5% discount rate, 138.5 quality-adjusted life-years (QALYs) would be gained at an extra cost of AUS$ 4.44 million and an incremental cost-effectiveness ratio of AUS$ 32,065 per QALY gained. Sensitivity and scenario analyses demonstrated that outcomes were most sensitive to assumptions around vaccine effectiveness, duration of protection in mothers, and disutility of unreported cases.

In conclusion, dTpa vaccination in the third trimester of pregnancy is likely to be cost-effective from a healthcare payer perspective in Australia.

## Introduction

Pertussis or whooping cough, caused by *Bordetella pertussis*, is a highly infectious bacterial respiratory infection affecting all ages. Despite national immunization programs, it causes significant morbidity and mortality, primarily in infants too young to be fully protected by vaccination.^1-3^

Annually around 140,000 pertussis cases are reported and 89,000 pertussis deaths are estimated worldwide,^4^ with the highest rates reported in Australia for over two decades (e.g., incidence in 2009 of 140 versus <10 per 100,000 in the United States of America, the United Kingdom (UK), Canada and China^5^); this can be partially explained by the national notification system and positive test reporting in Australia.^6^

Infants are at greatest risk of severe infection resulting in hospitalization and death,^2^ with most deaths among the youngest infants having received less than two doses of pertussis vaccine.^7^ Pertussis among adolescents and adults may present with atypical or milder symptoms,^8^^,^^9^ leading to under-diagnosis and under-reporting, and the risk of transmission to more vulnerable groups which could contribute to outbreaks in the population.^10^

Studies of pertussis-related intensive care unit (ICU) admissions in Australia and New Zealand between 1997 and 2014, including the 2009–2012 Australian epidemic, found that 1% of infants admitted to the ICU had pertussis, around half of whom required mechanical ventilation with some requiring invasive respiratory support, 4.8%-6.2% of pertussis ICU admissions resulted in death, among which 80%-87% were infants <4 months old.^1^^,^^3^ These infants were too young to have been protected by direct vaccination. The studies concluded more needs to be done to reduce the infant burden and significant healthcare costs (in excess of US$1 million [United States Dollars] per year).^1^^,^^3^

Vaccination reduces infection incidence by more than 95%.^2^ The 2016 nationally-funded pertussis program in Australia included DTPa (Diphtheria, Tetanus, acellular Pertussis) at 2, 4 and 6 months, followed by booster doses at 18 months and 4 years, and vaccination with reduced antigen dTpa at 12–13 years.^11^ Since March 2015, maternal dTpa vaccination in the third trimester of every pregnancy is recommended by the Australian Immunisation Handbook, but not funded nationally.^2^^,^^7^ Maternal vaccination allows passive transfer of antibodies to the fetus providing passive protection from the time of birth, as well as protection by preventing pertussis transmission from vaccinated mothers.^2^ In case-control and surveillance studies in the UK, maternal vaccination prevented 91 and 93% of laboratory-confirmed pertussis cases in infants <3 months old.^12–14^ Economic studies typically focused on prevention of pertussis in infants alone,^15–17^ as they are at greatest risk of severe consequences. However, important clinical and economic gains achieved from direct protection of mothers are often overlooked.

The objective of this study was to assess the cost-effectiveness of adding maternal dTpa vaccination against pertussis to the 2016 nationally-funded vaccination program in Australia, which includes infant and adolescent sequential doses of DTPa (*Infanrix*, GSK) at 2, 4, 6, 18 months (m) and 4 years (y) and a single dose of dTpa (*Boostrix*, GSK) at 12–13 years. The two vaccination strategies are further referred to as the maternal (ma) (m2-4-6-18,y4-13+ma) strategy and the 2016 (m2-4-6-18,y4-13) strategy.

## Results

Under base-case assumptions with 70% coverage, the maternal strategy resulted in 8,847 fewer symptomatic pertussis cases (i.e., 567 reported and 8,280 unreported cases), 422 fewer outpatient cases, 146 fewer hospitalizations (including 23 with complications) and 0.54 fewer deaths per year versus the 2016 strategy. The maternal strategy resulted in increased undiscounted vaccination costs (AUS$ 5.16 million per year) and treatment cost-savings from fewer reported pertussis cases (AUS$ 0.75 million per year). The undiscounted incremental cost of the maternal strategy was AUS$ 4.41 million per year. Adding maternal vaccination to the 2016 strategy led to a gain of 187.2 (undiscounted) quality-adjusted life-years (QALYs) per year, due primarily to preventing unreported symptomatic cases (+119.0 QALYs), infant deaths (+47.0 QALYs), and reported pertussis cases (+21.3 QALYs) (Table 1). Adding maternal vaccination to the 2016 vaccination strategy resulted in an additional discounted cost of AUS$ 4.44 million and 138.5 additional discounted QALYs gained per year, resulting in a cost per QALY gained of AUS$ 32,065 (Table 2).

**Table 1.** Undiscounted outcomes and costs (maternal versus 2016 strategy).

	2016 strategy (m2-4-6-18,y4-13)	Maternal strategy (m2-4-6-18,y4-13+ma)	Incremental
DTPa vaccinations	1,459,313	1,459,313	0
dTpa vaccinations	210,174	434,684	224,510
Symptomatic pertussis cases (total)	291,789	282,942	−8,847
Reported cases	24,063	23,496	−567
Hospitalized cases, no complications	987	864	−123
Hospitalized cases, with complications	180	157	−23
Outpatient cases	22,897	22,475	−422
Unreported cases	267,725	259,446	−8,280
Pertussis deaths	1.01	0.47	−0.54
Direct costs (undiscounted) (AUS$)			
DTPa vaccination	26,267,640	26,267,640	0
dTpa vaccination	4,833,996	9,997,720	5,163,724
Total vaccination costs	31,101,636	36,265,360	5,163,724
Hospitalized cases, no complications	4,048,606	3,545,815	−502,791
Hospitalized cases with complications	1,379,683	1,208,342	−171,341
Outpatient cases	4,053,714	3,978,941	−74,773
Total treatment costs	9,482,003	8,733,098	–748,905
Total direct costs	40,583,639	44,998,458	4,414,819
QALY loss (undiscounted)			
Deaths = LY loss	87.83	40.88	−46.95
Unreported cases	3,941.86	3,822.86	−119.00
Hospitalized cases, no complications	40.66	32.80	−7.86
Hospitalized cases with complications	7.47	6.02	−1.45
Outpatient cases	759.36	747.42	−11.94
Total QALY loss	4,837.18	4,649.98	–187.20

Due to rounding, some totals may not correspond with the sum of the separate values.

AUS$: Australian Dollars; DTPa: Diphtheria, Tetanus, acellular Pertussis; dTpa: reduced antigen diphtheria, Tetanus, acellular pertussis; LY: life-year; m2-4-6-18,y4-13: 2016 pertussis vaccination strategy, which includes infant and adolescent sequential doses of DTPa at 2, 4, 6, 18 months and 4 years and a single dose of dTpa at 12–13 years; m2-4-6-18,y4-13+ma: maternal strategy, which includes the addition of maternal dTpa vaccination to the 2016 strategy; QALY: quality-adjusted life-year.

**Table 2.** Discounted direct costs, QALYs and ICER (maternal versus 2016 strategy).

	2016 vs. NV strategy (m2-4-6-18,y4-13)	Maternal vs. NV strategy (m2-4-6-18,y4-13+ma)	Maternal vs. 2016
Direct incremental costs (5% discount) (AUS$)			
DTPa vaccination	26,267,640	26,267,640	0
dTpa vaccination	4,833,996	9,997,720	5,163,724
Total incremental vaccination costs	31,101,636	36,265,360	5,163,724
Hospitalized cases, no complications	−2,951,310	−3,440,116	−488,806
Hospitalized cases with complications	−1,005,747	−1,172,322	−166,575
Outpatient cases	−2,301,134	−2,368,843	−67,709
Total incremental treatment costs	−6,258,191	−6,981,281	−723,090
Total direct costs	24,843,445	29,284,079	4,440,634
QALYs gained (5% discount)			
Deaths	9.23	19.65	10.42
Unreported cases	666.42	774.59	108.17
Hospitalized cases, no complications	37.31	44.97	7.66
Hospitalized cases, with complications	6.86	8.27	1.41
Outpatient cases	574.62	585.44	10.82
Total QALYs gained	1,294.44	1,432.93	138.49
ICER (cost per QALY gained)			32,065

2016: 2016 pertussis vaccination strategy (m2-4-6-18,y4-13), which includes infant and adolescent sequential doses of DTPa at 2, 4, 6, 18 months and 4 years and a single dose of dTpa at 12–13 years; AUS$: Australian Dollars; DTPa: Diphtheria, Tetanus, acellular Pertussis; dTpa: reduced antigen diphtheria, Tetanus, acellular pertussis; ICER: incremental cost-effectiveness ratio; Maternal: maternal vaccination strategy (m2-4-6-18,y4-13+ma), which includes the addition of maternal dTpa vaccination to the 2016 strategy; NV: no vaccination; QALY: quality-adjusted life-year

**Figure 1.**
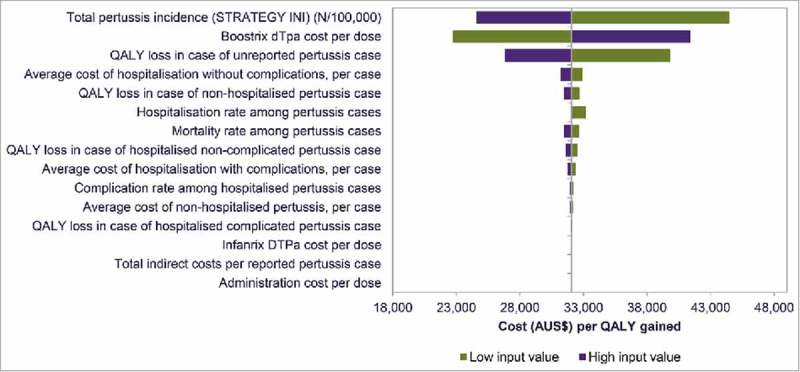
One-way sensitivity analysis on ICER. Fig. 1 shows the outcomes of the one-way sensitivity analyses (i.e. replacing the base-case input with a higher or lower input value) on the cost per QALY gained. The vertical line represents the base-case cost per QALY gained with the maternal versus 2016 strategy. AUS$: Australian Dollar; dTpa: reduced antigen diphtheria, Tetanus, acellular pertussis; DTPa: Diphtheria, Tetanus, acellular Pertussis; ICER: incremental cost-effectiveness ratio; QALY: quality-adjusted life-year; STRATEGY INI: strategy corresponding to incidence data.

Results of the one-way sensitivity analyses of varying key input parameters by plus or minus 25%, indicated that the results were most sensitive to variations in the total pre-vaccination pertussis incidence (incremental cost-effectiveness ratio [ICER] range AUS$ 24,608 – 44,494 per QALY gained), dTpa vaccine cost (ICER range AUS$ 22,744 – 41,387 per QALY gained), and disutility due to unreported symptomatic cases (ICER range AUS$ 26,827 – 39,846 per QALY gained). The model was less sensitive to other assumptions regarding costs and utility values (Fig. 1).

Results of the scenario analyses identified factors that led to lower ICERs compared with the base-case (ICER = AUS$ 32,065): i.e., when assuming the same disutility for unreported symptomatic cases as for reported outpatient cases (ICER = AUS$ 18,003), and, a longer duration of protection (10 years versus 5 years) for dTpa vaccinees other than mothers (ICER = AUS$ 24,436). The most important factors with an adverse impact on the cost-effectiveness of maternal vaccination were: assuming no benefit to mothers from maternal dTpa vaccination (ICER = AUS$ 190,713) and excluding unreported symptomatic pertussis cases from the analysis (ICER = AUS$ 146,488). Other factors that increased the ICER to a lesser extent included: reducing the duration of protection from dTpa to 1 year versus 5 years for mothers (ICER = AUS$ 70,864), and reducing the disutility for unreported symptomatic cases to 5%, 10% and 25% versus 50% of reported outpatient cases (ICER = AUS$ 107,962; AUS$ 85,481 and AUS$ 52,614 respectively).

The result of 1,000 probabilistic sensitivity analysis (PSA) simulations found that the maternal strategy was always more effective and more costly than the 2016 strategy (Fig. 2a). When assuming a willingness-to-pay threshold of AUS$ 45,000 per QALY as proxy, the probability of the maternal strategy being cost-effective was 93% versus the 2016 strategy (Fig. 2b).

**Figure 2a.**
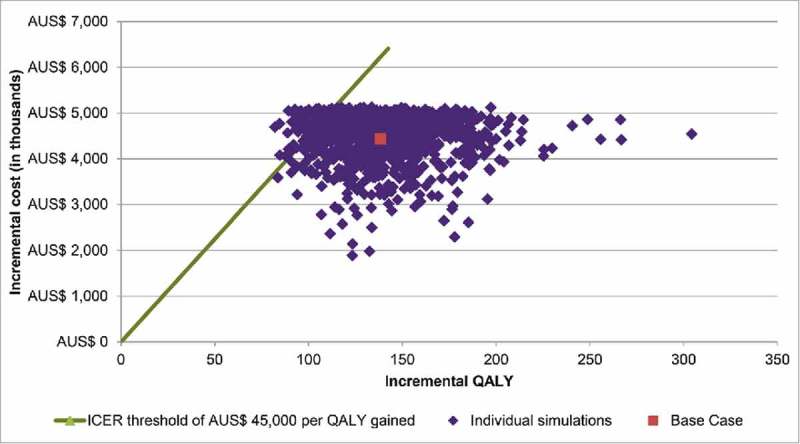
Probabilistic sensitivity analysis – cost-effectiveness plane. Figure 2a shows the results of varying the base-case inputs in the probabilistic sensitivity analysis.The points in this figure represent the incremental QALYs and costs gained in each simulation, which can be above or below the threshold line (i.e., AUS$ 45,000 per QALY gained). The central square represents the base-case outcomes. AUS$: Australian Dollar; ICER: incremental cost-effectiveness ratio; QALY: quality-adjusted life-year.

**Figure 2b.**
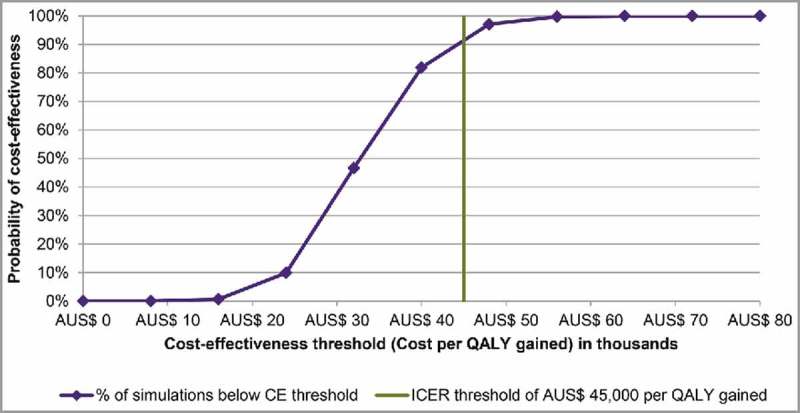
Cost-effectiveness acceptability curve (maternal versus 2016 strategy). In Figure 2b, the probability that the maternal strategy is cost-effective versus the 2016 strategy is determined by the percent of simulations that are below a given threshold. In this case, the maternal strategy was cost-effective in 93% of simulations at a threshold of AUS$ 45,000 per QALY gained. AUS$: Australian Dollar; CE: cost-effectiveness; ICER: incremental cost-effectiveness ratio; QALY: quality-adjusted life-year.

## Discussion

The addition of maternal vaccination to the 2016 pertussis strategy in Australia was predicted to prevent 8,847 more symptomatic pertussis cases (reported and unreported) annually as well as the associated complications, hospitalizations and outpatient visits, and one death every 22 months. Increased maternal vaccination program costs were partially offset by a reduction in treatment costs. Compared with the 2016 strategy, adding maternal vaccination was found to be a cost-effective strategy, providing increased health benefits (+138 discounted QALYs) at a cost of AUS$ 4.44 million (discounted at 5%). This resulted in a cost of AUS$ 32,065 per QALY gained in the base-case, within the cost-effectiveness range (up to AUS$ 45,000) as accepted by the Pharmaceutical Benefits Advisory Committee (PBAC) when it approved maternal pertussis vaccination. In sensitivity and scenario analyses, improvements in cost-effectiveness were driven by an increased reported pre-vaccination incidence of pertussis, increased disutility associated with unreported symptomatic cases, and longer duration of protection from dTpa. The maternal vaccination strategy was not considered cost-effective (at a AUS$ 45,000 threshold) when:

- the disutility associated with unreported symptomatic pertussis cases was less than 31.5% of the disutility of outpatient cases or
- unreported symptomatic cases were excluded from the analysis or
- dTpa had no effectiveness in mothers or infants or
- duration of protection in mothers was less than 2 years or
- dTpa cost was more than AUS$ 31.00.

In addition to notified cases, the literature from different countries suggest there is a significant burden due to clinically-significant pertussis-related illness that remains undiagnosed and/or unreported.^18^^,^^19^ These countries use different case notification definitions to Australia.^20^ The current analysis assumed that no costs were associated with unreported symptomatic cases. Assumptions around the number of unreported symptomatic cases and the disutility they experience due to pertussis had an important impact on the results. Data on under-reporting rates were limited, and are likely to vary across age groups, over time, by geographic region and vaccination practice. Our model applies symptomatic under-reporting rates obtained from a dynamic compartmental transmission model used in a previous PBAC submission, which was an adapted version (adapted to Australia) of a previously published model by de Vries *et al*. for the Netherlands.^21^ The symptomatic under-reporting rates represent less than 10% of the total under-reporting rate, in line with the estimates from de Vries *et al*. (Supplemental file 1). Assumptions were also made regarding disutility in unreported symptomatic cases (i.e., 50% of outpatient cases' disutility in the base-case), due to a lack of data. Disutilities for reported and unreported symptomatic pertussis cases were estimated from Lee *et al.*^22^ More recently, van Hoek *et al.*^23^ published a patient survey estimating the overall QALY loss to be 0.0972 for confirmed pertussis cases and 0.0365 QALY for household contacts with cough. These data suggest household contacts with cough have 38% of the QALY loss of confirmed pertussis cases. Although our base-case used a less conservative 50% disutility rate in unreported symptomatic cases, the scenario analysis found that maternal vaccination remained cost-effective as long as the rate was over 31.5%. Our model assumed a shorter duration of symptoms than van Hoek *et al*. resulting in lower overall QALY loss estimates (i.e., disutility of up to 0.0669 in hospitalized complicated cases and up to 0.0329 in unreported cases in our model, versus 0.0972 for confirmed pertussis cases and 0.0365 for household contacts with cough in van Hoek *et al*.^23^ In our model, symptoms lasted 80 days in reported and unreported pertussis cases, while van Hoek *et al*. reported 162 and 168 days for reported and unreported cases, respectively (also showing a similar duration of disutility in reported and unreported cases). The uncertainty related to the 50% reduction in QALY loss for unreported cases versus reported cases was not explored in probabilistic analyses.

Regarding the dTpa vaccine, the model assumed a direct protective effect in vaccinated mothers as well as a protective effect towards newborns waning over five years. Assuming no vaccine effectiveness in mothers had a large negative effect on the ICER. Assuming a one-year duration of protection in mothers, rather than five years, also increased the ICER. The duration of protection of acellular vaccines in adult populations was reported as being more than 10 years based on longitudinal antibody studies and mathematical modeling.^24^ Without established serological correlates of protection and without a clear understanding of the immune mechanisms associated with pertussis vaccination, uncertainty remains around duration of protection; however, there is consensus that high antibody levels, and by extrapolation immunity against disease, are likely to be maintained for at least five years post-vaccination.^24^

This analysis took a novel approach by assessing the benefits of a maternal vaccination program to both mothers and infants. Most previous cost-effectiveness analyses of maternal vaccination have only looked at the benefit in newborns, given the potentially severe consequences of pertussis infection in newborns. Maternal vaccination, however, can also prevent pertussis in mothers, and studies have demonstrated that pertussis vaccination can be cost-effective in adults.^25–28^ It is important, therefore, to include all direct benefits of maternal vaccination. A similar approach was also undertaken in a UK cost-effectiveness analysis of maternal vaccination.^29^ Another key strength of this analysis was the use of a population model, rather than more traditional modeling approaches, as it allowed consideration of a heterogeneous population rather than an average individual or cohort and simultaneous evaluation of multiple interventions which may be conflicting or complementary. The main limitations of the analysis were due to reliance on assumptions when data were lacking. There is uncertainty regarding pertussis incidence and several studies suggest high under-reporting rates, which has led to unreported cases being included in economic models.^30-32^ Unreported symptomatic pertussis cases were also included in this analysis, assuming they were less severe than reported cases by assigning much lower utility decrements to these cases. Moreover, no costs were assigned to unreported symptomatic cases, unlike a previous economic analysis which did consider associated medical costs.^32^ The inclusion of unreported symptomatic cases had a favourable impact on cost-effectiveness due to more avoided cases in protected individuals. Uncertainty associated with under-reporting and with the costs and disutilities of unreported symptomatic cases inevitably leads to uncertainty associated with the cost-effectiveness results. This is illustrated in the Tornado diagram showing that the incidence and the QALY loss associated with unreported cases are among the main drivers of cost-effectiveness results (Fig. 1). Another limitation of this analysis is the exclusion of herd effects (i.e., reduced transmission from vaccinated individuals) and of indirect costs prevented due to pertussis cases avoided (e.g., costs of parents missing working days due to pertussis infection of their infant), both of which are likely to improve the cost-effectiveness of the vaccination program.

Despite these uncertainties, the ICER for the addition of maternal pertussis vaccination to the 2016 schedule was within acceptable ranges, even within the conservative framework adopted. Additional factors that were not considered in this analysis but are likely to benefit pertussis-related health are herd effects from vaccination, cost-savings from unreported symptomatic cases, reduction in productivity losses (e.g., from parents caring for children with pertussis), and better health outcomes in the community from improved immunity to tetanus and diphtheria in the population.

## Conclusion

In conclusion, vaccination with dTpa during the third trimester of pregnancy represents a cost-effective intervention from the perspective of the healthcare payer in Australia.

## Methods

### Model structure

A static one-year cross-sectional population model was developed to assess the cost-utility of adding maternal dTpa vaccination to the 2016 pertussis program from the healthcare payer perspective (i.e., only direct health-related costs). The model assessed for each vaccination strategy the number of symptomatic pertussis cases (reported and unreported) and infant deaths occurring over a one-year period at steady-state (i.e., a hypothetical future year with vaccination at maximum coverage for a sufficiently long time to achieve its full impact in the population).^33-35^ Direct costs were attributed to vaccination and reported pertussis cases. QALYs lost were attributed to morbidity and mortality of reported and unreported symptomatic pertussis cases. This approach to modeling vaccination strategies versus more conventional longitudinal state-transition cohort methods (i.e., Markov or decision tree models) has been previously described^33^ and used adequately to model other infectious diseases.^35^

**Figure 3.**
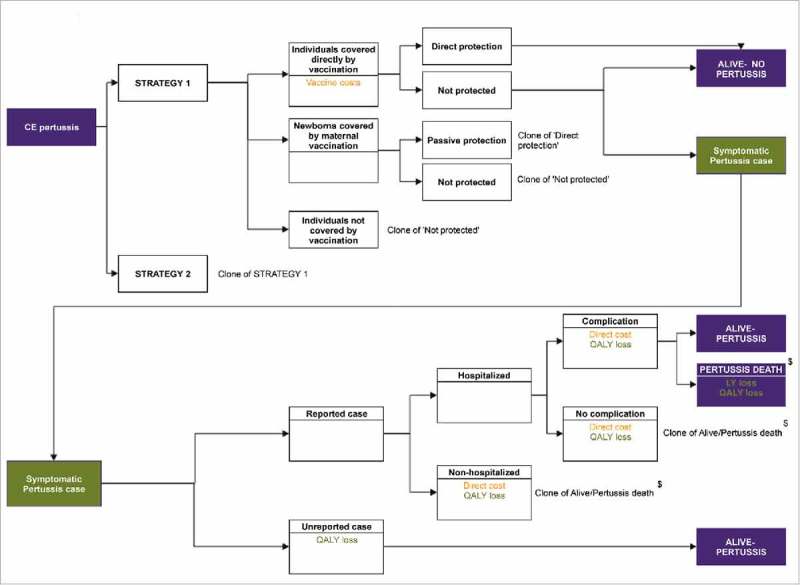
Pertussis model structure. Fig. 3 shows the pertussis health-economic model states through which subjects can progress to compare the cost-effectiveness of strategy 1 (2016 vaccination strategy) versus strategy 2 (maternal strategy). ^$^ The same mortality rate, i.e. the overall age-specific mortality rate in case of reported pertussis, was assigned to all reported cases. No distinction was made between mortality in non-hospitalized or hospitalized cases in the model. CE: cost-effectiveness; LY: life-year; QALY: quality-adjusted life-year; VE: vaccine efficacy.

The current model was built in MS Excel 2010 and the structure is demonstrated in Fig. 3. The population was split by age in months for 0 to 23 months, and by single year for ages 2 to 99 years.

### Epidemiological and demographic data

The impact of each vaccination strategy on pertussis incidence was determined by a reduction in pertussis incidence compared with a strategy without pertussis vaccination, considering direct vaccination protection and passive protection of infants from maternal vaccination. The ‘pre- vaccination’ pertussis incidence was retrospectively estimated by removing the effect of vaccination from available age-specific reported pertussis incidence: using vaccination strategies data in place at the time, the effect of vaccination was removed in each age group based on the coverage, vaccine efficacy at the respective age taking into account the time since vaccination, the duration of vaccine efficacy and waning of the vaccinations incorporated in the available incidence data. To calculate the incidence without vaccination, the following formula was used: Incidence incorporating vaccinations / (1 – Vaccine efficacy * Coverage). Starting from the calculated incidence rates without vaccination, the model then calculated the incidence rates with the maternal dTpa and the 2016 pertussis vaccination strategies, again based on the coverage, vaccine efficacy and duration of vaccine efficacy of the vaccinations included in these respective strategies. To calculate the incidence with vaccination, the following formula was used: Incidence without vaccination * (1 – Vaccine efficacy * Coverage).

The intermediate step of retrospectively estimating the incidence data without vaccination was performed to allow for correctly discounting costs and disutilities of avoided cases (See paragraph on discounting) back to the time of vaccination. This methodology also improved the clarity of the calculations of incidence rates in the population per vaccination strategy, given the complexity of the potential overlap of protection from consecutive vaccinations given to the same individual due to duration of vaccine efficacy. Three rules were applied in the model with regards to the impact of consecutive vaccinations. (1) Maximum overlap of coverage was assumed when different vaccines targeted the same age-groups. For example in the maternal strategy: 70% of mothers were vaccinated, therefore 70% of newborns were protected due to maternal vaccination. From two months of age, 91% of infants received their primary vaccination; the model assumed 70% of infants were protected by both maternal vaccination (passive protection) and the first dose of primary vaccination, while 21% of infants were only covered by the first dose of primary vaccination. (2) In a population protected by multiple vaccines, the maximum current protection level resulting from the respective vaccines was assigned. In the maternal strategy example; 70% of infants protected by both maternal vaccination and the first dose of primary vaccination were assigned a vaccine efficacy of 86% (i.e., the protection level at the age of 3 months based on waning of the 91% vaccine efficacy of maternal protection at birth) since this vaccine efficacy level was higher than the 60% vaccine efficacy of the first primary vaccination with DTPa. (3) The population that was not mutually covered was assigned the protection level of the vaccine with higher coverage. In the example, 21% of the population was assigned a vaccine efficacy of 60%.

Available pertussis incidence rates reported by the National Notifiable Diseases Surveillance System (NNDSS) were not stratified by month of age for infants, therefore a consolidated analysis with data for ages 0 to 11 months from 1999–2008 was used.^20^ Disease burden in subjects ≥1 year of age was estimated from the NNDSS^36^ using average data from 2008–2014, which incorporated vaccination strategies in place at the time (e.g., to account for the fact that only one booster DTPa dose was used at the time, the coverage of the DTPa booster was set to 50%), improved testing, diagnosis and reporting, recent epidemics, but not the impact of maternal vaccination programs. An age-specific multiplication rate was applied to reported incidences to account for under-reporting of symptomatic pertussis, which was assumed to be low in infants and children (Table 3). Under-reporting in age groups ≥1 year of age was estimated from Australian seroepidemiology survey data^37^ and a compartmental dynamic transmission model presented in the *Infanrix* (GSK) 18-month booster dose submission to the PBAC.^38^ The rate of under-reporting for pertussis was calculated by comparing the results of the seroincidence study by Campbell *et al*.^37^ and reported pertussis notifications by age from the NNDSS for Australia (Supplemental file 1).^36^ In our model, solely symptomatic under-reporting rates were applied, representing less than 10% of the overall under-reporting rates, in line with de Vries *et al*. who similarly estimated symptomatic cases to represent approximately 10% of all pertussis cases.

**Table 3.** Symptomatic pertussis incidence (Annual, per 100,000) and hospitalization rate by age group.

Age group	Reported incidence^20^^,^^36^ × under-reporting factor	Total incidence with vaccination	Calculated incidence without vaccination^£^	Hospitalization rate in reported cases
0 – <1 months	134 × 2	268	268	100%^20^
1 – <2 months	245 × 2	490	490	
2 – <3 months	232 × 2	464	464	
3 – <4 months	150 × 2	300	661	
4 – <5 months	118 × 2	236	515	
5 – <6 months	68 × 2	136	375	
6 – <7 months	50 × 2	100	272	39.40%^41^
7 – <8 months	50 × 2	100	552	
8 – <9 months	50 × 2	100	532	
9 – <10 months	50 × 2	100	514	
10 – <11 months	50 × 2	100	496	
11 – <12 months	50 × 2	100	480	
1 – <5 years	196 × 3	587	2,128	7.40%^41^
5 – <10 years	272 × 3	817	1,549	1.50%^41^
10 – <15 years	215 × 3	644	1,106	1.87%^41^
15 – <20 years	63 × 20	1,258	1,553	
20 – <25 years	44 × 20	887	887	
25 – <30 years	47 × 20	947	947	2.10%^41^
30 – <35 years	68 × 20	1,359	1,359	
35 – <40 years	94 × 20	1,884	1,884	1.70%^41^
40 – <45 years	104 × 20	2,087	2,087	
45 – <50 years	91 × 20	1,822	1,822	2.80%^41^
50 – <70 years	92 × 15	1,379	1,379	4.50%^41^
70 – <90 years	82 × 10	818	818	11.40%^41^
≥90 years	48 × 10	475	475	11.40%^41^

† Total = reported and under-reported incidence with vaccination strategies in place at the time ^£^ Calculated incidence considering the coverage of the vaccination strategy in place at the time, and the vaccine efficacy, duration of protection and waning

The population age distribution and age distribution of mothers were retrieved from the Australian Bureau of Statistics (ABS).^39^ The model assumed a mortality rate of 0.4% for reported cases in infants <6 months old, based on the Australian Technical Advisory Group on Immunisation (ATAGI) pre-PBAC advice in 2015^40^ and no pertussis-related mortality in any other age groups. Based on Foxwel *et al*. the hospitalization rate for children <6 months old was assumed to be 100%.^20^ Hospitalization rates in other age groups were from Clarke *et al.* (Table 3).^41^ Hospitalized cases with complications (i.e., 15.14% for all ages) were estimated from Australian Institute of Health and Welfare (AIHW) 2013–2014 hospitalization separation statistics, using Australian-Refined Diagnostic-Related Group (AR-DRG) codes E70A and E70B.^42^

### Vaccine inputs

The model assumed the DTPa vaccine was given for the primary vaccination series (2, 4, 6 months) and booster doses at 18 months and 4 years, while 13 year olds and pregnant women received dTpa.

Protection against pertussis infection in each strategy was provided by age-specific vaccination coverage and direct vaccine efficacy waning linearly over time to become 0% at the end of the vaccine efficacy duration (Table 4). Maximum vaccine efficacy of dTpa in mothers (92%) was estimated from the APERT study^43^ waning over five years. While the literature supports antibody protection for at least 10 years,^44^ a five-year duration of protection was selected to account for mothers who are re-vaccinated with subsequent pregnancies. Passive vaccine protection in infants due to maternal vaccination was estimated from UK observational data showing high levels of protection (91%) in infants up to three months old.^12^ Vaccine protection was assumed to start at birth (91% vaccine effectiveness), based on transmission of immunoglobulins from the mother, and to wane over five years, resulting in a lower vaccine effectiveness in two- and three-month old infants than the UK study. Protection from primary DTPa vaccination began at two months of age, and by seven months of age the waning protective effect of maternal vaccination was completely superseded by the higher level of protection from primary DTPa vaccination. The Australian clinical recommendation is to vaccinate mothers for every pregnancy regardless of the time between subsequent pregnancies.^7^^,^^40^ The model includes vaccination at each pregnancy and implicitly assumes all newborns within a 5-year time horizon have an unimmunized mother, i.e., it assumes that vaccinated mothers weren't vaccinated against pertussis during the previous 5 years. Consequently, the effect of maternal vaccination was assigned without assuming overlap of protection from consecutive maternal vaccinations. Coverage with DTPa (91%) was estimated from National Centre for Immunisation Research & Surveillance (NCIRS) data for 2014.^11^ Coverage with dTpa was estimated at 72% for adolescents and 70% for pregnant women, from ATAGI advice on uptake in State and Territory initiatives and UK national vaccination experience.^12^ Infants born to vaccinated mothers and thereafter vaccinated with DTPa were assigned the maximum of either protection level.

**Table 4:** Vaccine inputs and assumptions, disutilities, resource use and costs

Vaccine inputs and assumptions												
Vaccine by age	Vaccine efficacy					Waning period					Coverage	
2 months, DTPa	60%					10 years					91%^11^	
4 months, DTPa	70%					10 years					91%^11^	
6 months, DTPa	90%					10 years					91%^11^	
18 months, DTPa	90%					10 years					91%^11^	
4 years, DTPa	90%					10 years					91%^11^	
13 years, dTpa	92%^40^					5 years					72%^12^	
Maternal, dTpa	92% in mothers^40^					5 years in mothers					70%^12^	
	91% in infants^12^					5 years in infants					70%^12, 41^	
Age-specific disutility associated with pertussis cases												
Disutility by age	Outpatient cases^a^		Hospital^b^, no complications					Hospital^b^, with complications				Unreported cases^c^
<1 year	0.30^e^		0.42^d^					0.42^d^				0.15
1-4 years	0.28^e^		0.39^e^					0.39^e^				0.14
5-9 years	0.25^e^		0.36^e^					0.36^e^				0.13
Adolescents (10-19 years)	0.22		0.33					0.33				0.11
Adults (20+ years)	0.15		0.19					0.19				0.08
Used disutility for: ^a^mild cough, ^b^severe cough, ^c^50% of outpatient cases, ^d^infant respiratory complication, ^e^estimated based on linear interpolation												
Resource use and costs												
		Direct cost (AUS$)							Assumptions and sources			
*Infanrix* (GSK), DTPa, per dose		18								Approximate NIP price		
*Boostrix* (GSK), dTpa, per dose		23								Approximate NIP price		
Inpatient cases, no complications		4,104								AR-DRG item E70A and E70B^42^		
Inpatient cases, with complications		7,683										
Unreported cases		0								Assumption		
Outpatient cases		195.40 (<1 year old)			177 (≥1 year old)					See utilization assumptions below		
		Unit cost (AUS$)		Utilization						Sources		
GP consultation		37.05		3.7			3.7			MBS 23^44,49^		
Specialist visit		85.55		0.1			0.1			MBS 104^44,49^		
Culture		22.00		9.6%			1.6%			MBS 69303^44–46^		
Serology		28.65		59.7%			16.6%			MBS 69494^44–46^		
PCR		15.65		8.7%			64.4%			MBS 69384^44–46^		
Medical treatment <1/y		29.18		1			N/A			PBS 9192T^48,49^		

AR-DRG: Australian-refined Diagnosis Related Groups; AUS$: Australian Dollar; DTPa: Diphtheria, Tetanus, acellular Pertussis; dTpa: reduced antigen diphtheria, Tetanus, acellular pertussis; GP: general practitioner; N/A: not applicable; NIP: National Immunization Program; PCR: Polymerase chain reaction; y: year

### Utilities and costs

Disutilities, for reported and unreported symptomatic pertussis cases, were estimated from a time-trade-off study^22^ and applied to the duration of disease (with or without hospitalization or complications) to estimate the QALY loss per case. Unreported symptomatic cases were assumed to accrue 50% of the outpatient case disutility. Disease duration was assumed to be 80 days (<1-year-olds), 62 days (1-to-19-years-olds) and 68 days (≥20-years-olds)^26^^,^^27^ according to literature from other countries with similar development levels. Disease duration was assumed to be the same for reported and unreported cases. The average hospital length of stay was 3.7 days with complications and 1.98 days without.^45^ Future QALY loss due to pertussis deaths was calculated from life-expectancy at the time of death and assumed to be equal to future life-years (LYs) lost (i.e., no further utility loss was applied). Life expectancy at birth (i.e., 86.75 years undiscounted) was retrieved from the Australian Bureau of Statistics.^46^

The vaccine prices per dose of *Infanrix* (GSK) and *Boostrix* (GSK) are listed in Table 4. Average hospitalization costs with and without complications and comorbidities were estimated from National Hospital Cost Data Collection (Round 17) AR-DRG items E70A and E70B, respectively.^45^ Reported outpatient-care costs were based on expected general practioner (GP) and/or specialist consultations, testing and treatment resource use, as described in Table 4.^47–51^ No costs were attributed to unreported symptomatic pertussis cases.

### Discounting

A temporal delay exists between the age at which a vaccine is given and the ages at which the impact of the vaccination is experienced, i.e. when pertussis cases are avoided with corresponding impact on health and costs. According to economic guidelines, future cost consequences and health consequences from an intervention should be discounted. Therefore, although the model considers only a 1-year time horizon at steady-state, discounting is applied on costs and QALYs to reflect the fact that the intervention required to achieve the steady-state took place in an earlier time period (at younger age). Discounting was applied to costs and outcomes, as per PBAC guidelines, at 5% per annum. Discounting cannot be performed on the pertussis cases taking place since these are not linked to a vaccination. Therefore, discounting was only applied to QALY gains and cost-savings of avoided pertussis cases due to direct vaccine efficacy or due to passive protection in newborns after maternal vaccination, based on the time between vaccination and the avoided event. In addition, the model attributes future QALYs gained to each fatal case avoided due to vaccine efficacy. These future QALYs are discounted to the time of death. The resulting discounted QALYs gained at the time of death are then additionally discounted to the time of vaccination, based on the time between vaccination and avoided death. Vaccination costs were immediate and therefore not discounted. Discounted incremental costs and outcomes were first calculated for each strategy versus no vaccination, then these differences were compared to establish the incremental discounted costs and outcomes for the maternal versus the 2016 strategy.

### Uncertainty analyses

One-way sensitivity analyses estimated the impact on the ICER of varying key input parameters by plus or minus 25% (i.e., pertussis incidence, hospitalization, complication and mortality rates due to pertussis, treatment and vaccination costs, and, QALY losses associated with outpatient, hospitalized, complicated and unreported symptomatic pertussis cases).

In scenario analyses, uncertainties around input parameters were tested by: 1) excluding unreported symptomatic pertussis; 2) excluding maternal protection from dTpa; 3) excluding infant protection from dTpa; 4) changing the disutility of unreported symptomatic cases to 5%, 10% or 25% versus 50% of the reported outpatient disutility; 5) changing the duration of dTpa protection. The Probabilistic Sensitivity Analysis (PSA), using assigned distributions applied to the same parameters as included in the one-way sensitivity analyses, estimated incremental QALYs and costs for the maternal versus 2016 strategy from 1,000 simulations (Table 5).

**Table 5.** Probabilistic sensitivity analyses distribution.

Parameters	Distributions	Calculation method
Rates		
Pertussis incidence	Beta	N = 100,000^¥^ α = n, β = N-n
Mortality rates due to pertussis (infants <6 months)	Beta	N = 100^¥^ α = n, β = N-n
Hospitalization rate among pertussis cases	Beta	N = 100^¥^ α = n, β = N-n
Complication rate among hospitalized pertussis cases	Beta	N = 100^¥^ α = n, β = N-n
Disutilities		
Outpatient case	Beta	SE = 95%CI/(2*1.96)° α = mean*((mean*(1-mean)/SE^2^)-1) β = (α–mean*α)/mean
Inpatient case, no complications	Beta	SE = 95%CI/(2*1.96)° α = mean*((mean*(1-mean)/SE^2^)-1) β = (α–mean*α)/mean
Inpatient case, with complications	Beta	Equal to disutilities related to inpatient case, no complications
Unreported case	Beta	Equal to 0.5 of outpatient cases
Durations		
Average duration of illness per case of pertussis	Gamma	SE = 95%CI/(2*1.96)° α = mean^2^/SE^2^, β = SE^2^/mean
Average hospital length of stay with complications	Gamma	SE = 95%CI/(2*1.96)° α = mean^2^/SE^2^, β = SE^2^/mean
Average hospital length of stay without complications	Gamma	SE = 95%CI/(2*1.96)° α = mean^2^/SE^2^, β = SE^2^/mean
Costs		
Vaccine price	Fixed	
Outpatient case	Gamma	Assumption SE = mean^52^ α = mean^2^/SE^2^, β = SE^2^/mean
Inpatient case, no complications	Gamma	Assumption SE = mean^52^ α = mean^2^/SE^2^, β = SE^2^/mean
Inpatient case, with complications	Gamma	Assumption SE = mean^52^ α = mean^2^/SE^2^, β = SE^2^/mean

¥ Sample size (N) is an assumption; °95% CI is based on an assumption = mean±25%

CI: confidence interval; N: sample size; n: number of events of interest; SE: standard error of the mean

## Declarations

### Trademark statement

Boostrix and Infanrix are trade marks owned by or licensed to the GSK group of companies.

## Supplementary Material

KHVI_A_1474315_Supplemental.zip
