# Supplementary material for: Is adding maternal vaccination to prevent whooping cough cost-effective in Australia?
Source: Hum Vaccin Immunother. 2018 Jun 22;14(9):2263–73. doi: 10.1080/21645515.2018.1474315 (PMC6183273; doi:10.1080/21645515.2018.1474315)
Supplement: KHVI_A_1474315_Supplemental.zip [file khvi-14-09-1474315-s001.zip › KHVI_A_1474315_Supplemental 1.docx]

# Supplemental file 1: Under-reporting rates

A dynamic transmission model was used in a previous submission to the PBAC supporting the introduction of an 18-month pertussis booster dose.^38,54^ The model was adapted to Australia from a previously-published Dutch model^21^ and calibration methodology.^55^ The dynamic transmission model used a Susceptible, Infective and Removal (SIR) structure to estimate the total number of expected pertussis cases in Australia.

The transmission model included 3 different types of infection, symptomatic reported, symptomatic unreported and asymptomatic. The relative infectiveness of each infection type was sourced from de Vries *et al*.^21^ The model incorporated vaccine waning assumptions in line with published literature and utilized the contact matrix for Great Britain published by Mossong *et al*.^56^

The model was calibrated and the overall incidence of pertussis by age closely matched the published seroincidence results.^37^ The rate of under-reporting for pertussis was calculated by comparing the results of the seroincidence study by Campbell *et al*.^37^ and reported pertussis notifications by age from the NNDSS for Australia.^36^

The most recent year with reliable and available data for both seroincidence and pertussis notifications was 2007. Using data from 2007 in the base-case assumes that overall pertussis infection was at an equilibrium state in that year. Seroincidence data for 2007 from Campbell *et al*.^37^ are presented in Appendix Figure 1 as a solid blue line alongside pertussis notifications. Pertussis notifications are publicly available as they are reported to the NNDSS. The data for 2007 is displayed in Appendix Figure 1 as a red dotted line.

Seroincidence data from Campbell *et al*.^37^ and notification rates from the NNDSS^36^ were used directly in the dynamic compartmental transmission model. The under-reporting rate was calculated by dividing the total number of seropositive individuals per 100,000 (defined as immunoglobulin G (IgG) anti-Pertussis Toxine (PT) ≥62.5 EU/ml indicating infection in the past 12 months^57^) by the reported incidence rate per 100,000. This comparison indicates that one out of every 182 cases of pertussis in 2007 was reported in Australia.

Annual incidence rates under the status quo (for symptomatic and asymptomatic cases) closely reflected the seroincidence data from Campbell *et al*.^37^ after calibration had been completed, as seen in Appendix Figure 2. The seroincidence data from Campbell *et al*.^37^ can be seen in Appendix Figure 2, as a green line, where the calibrated incidence is represented as a blue line.

Whereas the total under-reporting rate across all age groups was 182 in 2007, the maternal model only addresses the symptomatic under-reporting rates which were obtained from the dynamic compartmental transmission model and range from 2 to 20 across different age groups (Table 3). Compared to the total under-reporting rate, the proportion of symptomatic under-reporting is in line with the proportion of symptomatic underreported cases according to de Vries *et al*., who estimated that approximately 10% of all pertussis cases (reported or unreported, symptomatic or asymptomatic) are symptomatic (I3 out of Total in Figure 3 of de Vries *et al*.). ^21^

The symptomatic under-reporting rate assumed in the maternal model was a low rate in infants and children <15 years, and up to a maximum of 20 in older age groups.

# Figure Legends

Appendix Figure 1. Seroincidence and notifications for pertussis in 2007

Appendix Figure 2. Calibrated incidence by age
