# Supplementary figures and images for: Is adding maternal vaccination to prevent whooping cough cost-effective in Australia?
Source: Hum Vaccin Immunother. 2018 Jun 22;14(9):2263–73. doi: 10.1080/21645515.2018.1474315 (PMC6183273; doi:10.1080/21645515.2018.1474315)

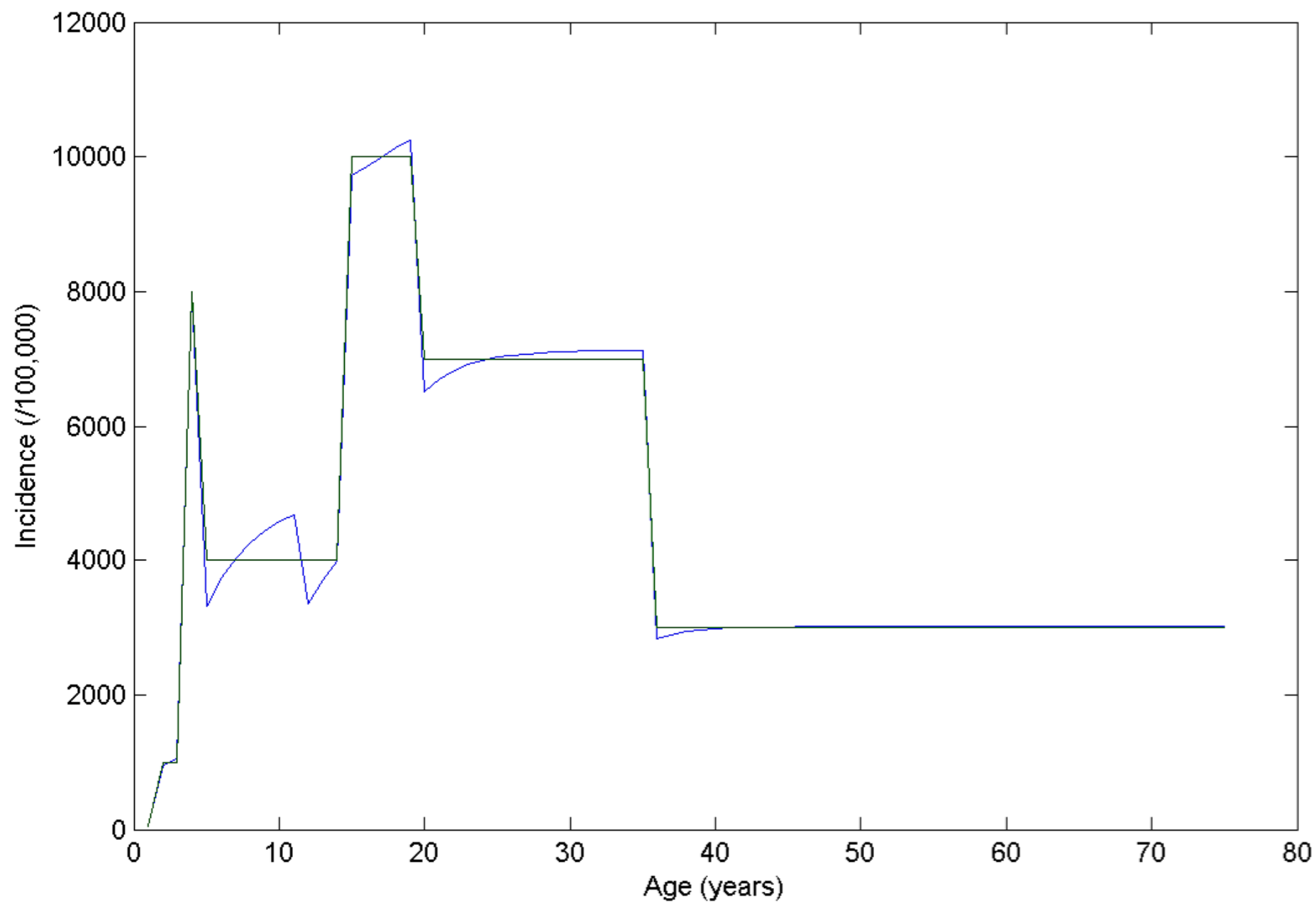

Supplement: KHVI_A_1474315_Supplemental.zip [file khvi-14-09-1474315-s001.zip › KHVI_A_1474315_Supplemental 3.pdf]

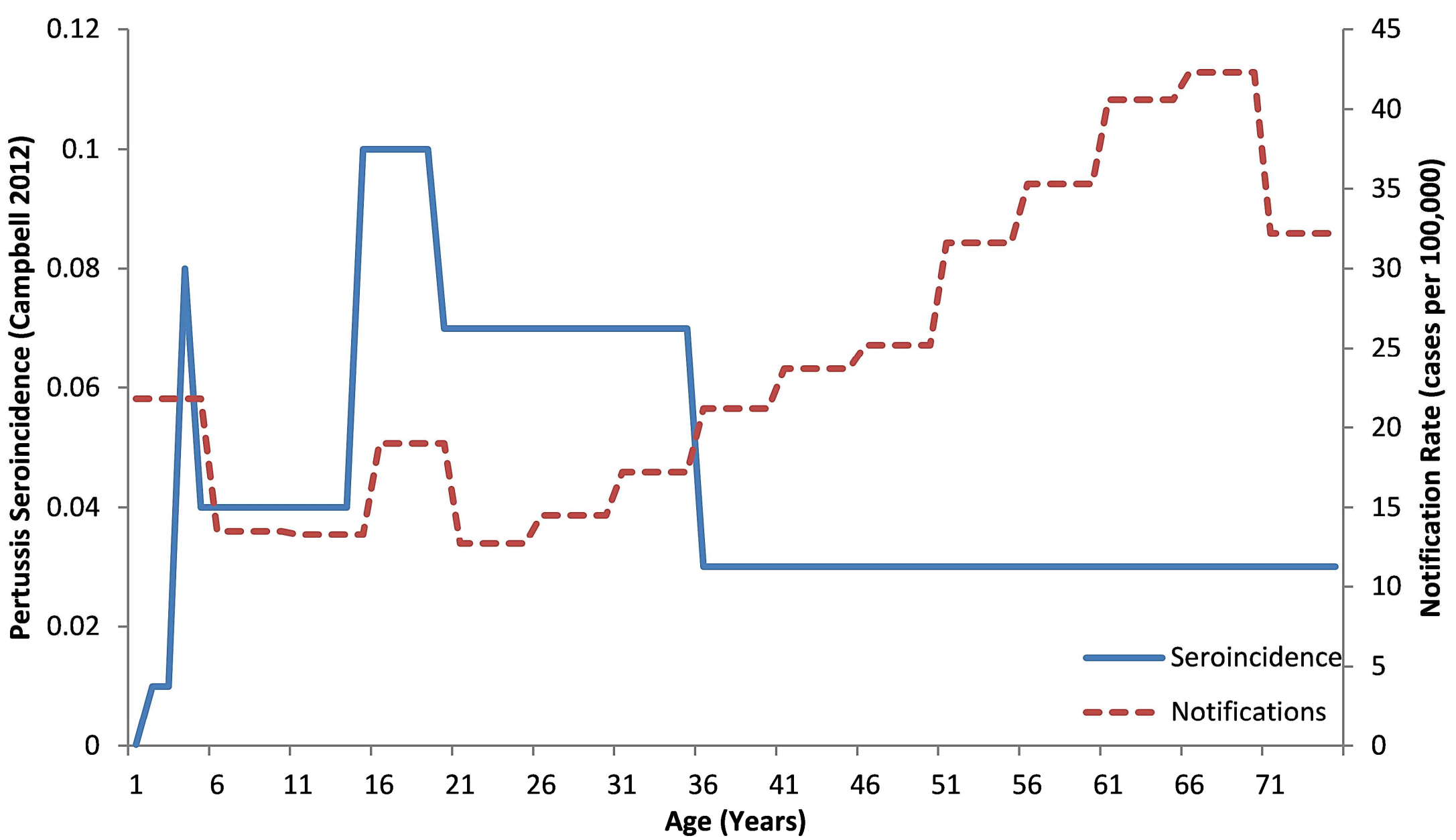

Supplement: KHVI_A_1474315_Supplemental.zip [file khvi-14-09-1474315-s001.zip › KHVI_A_1474315_Supplemental 4.pdf]
